# The biochemical basis of disease

**DOI:** 10.1042/EBC20170054

**Published:** 2018-12-03

**Authors:** Alastair J. Barr

**Affiliations:** Department of Biomedical Science, University of Westminster, London, U.K.

**Keywords:** cardiovascular disease, cancer, metabolic disorders, molecular basis of health and disease, microbiology, neurodegeneration

## Abstract

This article gives the reader an insight into the role of biochemistry in some of the current global health and disease problems. It surveys the biochemical causes of disease in an accessible and succinct form while also bringing in aspects of pharmacology, cell biology, pathology and physiology which are closely aligned with biochemistry. The discussion of the selected diseases highlights exciting new developments and illuminates key biochemical pathways and commonalities. The article includes coverage of diabetes, atherosclerosis, cancer, microorganisms and disease, nutrition, liver disease and Alzheimer’s disease, but does not attempt to be comprehensive in its coverage of disease, since this is beyond its remit and scope. Consequently there are many fascinating biochemical aspects of diseases, both common and rare, that are not addressed here that can be explored in the further reading cited. Techniques and biochemical procedures for studying disease are not covered in detail here, but these can be found readily in a range of biochemical methods sources.

## Diabetes

### Introduction

Diabetes mellitus is a condition in which the body is unable to control blood glucose levels adequately, resulting in high blood glucose levels (hyperglycaemia). Symptoms include frequent urination due to the osmotic effect of excess glucose in the urine, thirst due to loss of fluids and weight loss. Possible long-term complications of diabetes if blood glucose has been poorly controlled include cardiovascular disease (such as atherosclerosis and stroke) and damage to nerves, the kidney and eyes, which can potentially lead to blindness. Diabetes is a major health problem with an estimated 425 million people affected worldwide, and these numbers are predicted to rise. The rise in numbers is associated with an increase in obesity in the population and treating the complications is a major healthcare cost. In the U.K., some estimates predict the cost could reach 17% of the NHS budget.

Most people will be familiar with the classification of diabetes into the two main forms, Type 1 and Type 2; however, it is increasingly clear that there are in fact several different types of diabetes, some of which overlap to some extent. Recent research analysing nearly 15000 diabetics showed they could be clustered into five distinct groups based on specific biomarkers^1^ of the condition, which is significant because this better classification system may lead to improved treatment strategies in the future. Type 1 diabetes is an autoimmune disease in which cells of the body’s immune system cause destruction of insulin secreting β-cells in the pancreas, leading to a deficiency of insulin production. There are typically antibodies against key pancreatic proteins involved in insulin storage and secretion. It is a relatively rare form of the disease affecting 5–10% of diabetics, which is usually diagnosed in childhood and is not associated with excess body weight. Type 2 diabetes is the more common form of the disease, affecting 90–95% of diabetics, and is characterised by a loss of ability to respond to insulin (i.e. there is insulin resistance, also termed as insulin insensitivity). At diagnosis, individuals are typically over 30 years old, overweight, have high blood pressure and an unhealthy lipid profile (referred to as the metabolic syndrome). Established disease is associated with hypersecretion of insulin, but this is still inadequate to restore normal blood glucose levels, and the condition may progress towards insulin deficiency. The causes of diabetes are thought to be a combination of genetic and environmental factors, and it is recognised that being overweight is a strong risk factor for developing Type 2 diabetes.

### Insulin action

In healthy individuals, blood glucose levels range between 3.5 and 5.5 mmol/l before meals. This range is maintained by the actions of hormones (primarily insulin and glucagon, but also adrenaline, cortisol and growth hormone) which control the production and uptake of glucose, levels of glycogen (the stored form of glucose), and fat and protein metabolism, as required following meals, during fasting and exercise. Both insulin and glucagon are polypeptides produced by the pancreas (β-cells – insulin; α-cells – glucagon).

Insulin is secreted in response to an increase in blood glucose levels and its overall effect is to store chemical energy by enhancing the uptake and storage of glucose, amino acids and fats; consequently reducing blood glucose levels, via actions on liver, muscle and adipose tissue (specifically adipocytes – fat cells). Glucagon, on the other hand, via a complex interplay with other hormones and the nervous system increases blood glucose by stimulating the breakdown of glycogen, fat and protein. When blood glucose is high, after a meal for example, insulin acts on the liver to decrease glucose synthesis (gluconeogenesis), increase glucose utilisation (glycolysis) and increases glycogen synthesis (glycogenesis). When the storage capacity for glycogen is reached, insulin increases synthesis of fatty acids (lipogenesis), via acetyl CoA as an intermediate, which is then exported for triglyceride synthesis in adipocytes. In muscle, insulin stimulates uptake of glucose, by recruiting the glucose uptake transporter type 4 (GLUT-4), and enhances glycogen synthesis and glycolysis. In adipose tissue, there is facilitated uptake of glucose which is metabolised to glycerol and subsequently used together with fatty acids to synthesise triglycerides. Insulin also inhibits pathways involved in lipolysis. In addition, insulin increases amino acid uptake and protein synthesis in muscle and is considered an anabolic hormone (i.e. one that builds up organs and tissues).

**Figure 1:**
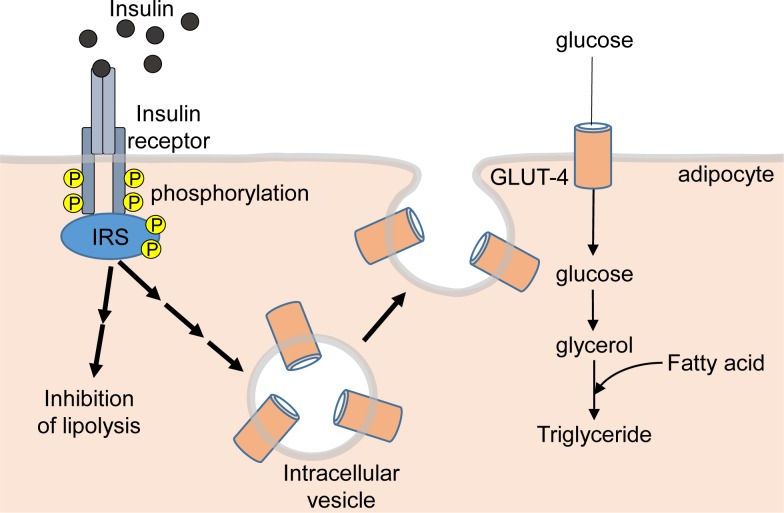
Insulin signalling in an adipocyte Abbreviation: P, phosphorylation on tyrosine.

At the biochemical level, insulin produces its effects by binding to the insulin receptor – a cell surface glycoprotein composed of two extracellular α subunits and two β subunits that span the membrane (Figure 1). The receptor has tyrosine kinase activity (i.e. enzyme activity that catalyses the transfer of a phosphate group from ATP to a tyrosine amino acid within a protein, also known as tyrosine phosphorylation). Binding of insulin to the receptor initially causes tyrosine phosphorylation of the receptor itself, and then phosphorylation of intracellular proteins termed as insulin receptor substrate (IRS)-1 and IRS-2, followed by a complex series of intracellular signalling events involving many other kinases that lead to the physiological changes in carbohydrate, fat and protein metabolism discussed above via changes in gene expression and the activity of metabolic enzymes. The effects of insulin on glucose uptake are mediated via the glucose transporter GLUT-4, which is stored in intracellular vesicles in an inactive state, and insulin stimulates the movement of these vesicles to the plasma membrane where GLUT-4 becomes inserted into the membrane forming a pore that allows glucose uptake into the cell (Figure 1).

### Disease complications and ketoacidosis

Many of the longer term complications of diabetes involve effects on both large arteries (macrovascular) and small arteries and capillaries (microvascular). High blood glucose leads to proteins and lipids becoming modified in a non-enzymatic process by exposure to sugars, forming advanced glycation end products that have been implicated in the disease process. Oxidative stress and damage to the vascular endothelium lining blood vessels is also involved. One of the diagnostic tests for diabetes involves measuring levels of glycated haemoglobin (HbA_1c_) from red blood cells. This is a valuable test because it gives an assessment of the average plasma glucose concentration over months, because of the 120 days lifespan of a red blood cell, and it also gives an indication of how effective treatment has been.

An acute serious life-threatening condition associated with untreated Type 1 diabetes is diabetic ketoacidosis. It develops in the absence of insulin, during which there is increased glucose production by the liver but because of the absence of insulin cells in the periphery, such as muscle cells, are unable to take-up the glucose and use it. The consequent high blood glucose levels results in the kidneys filtering and removing it from the body in urine. This is associated with osmotic diuresis (loss of fluids and electrolytes) and dehydration. As an alternative energy source, triglycerides (fats) from adipose tissue are broken down to free fatty acids and taken up by the liver. Here they are converted into acetyl CoA which is the precursor for formation of ketones (acetoacetate, β-hydroxy-butyrate and acetone) within mitochondria. These are referred to as ketone bodies and released into the blood and are detectable in the breath giving a distinctive smell similar to that of acetone or pear drops. Release of ketones into the blood causes a drop in pH (acidosis) and the body tries to compensate by hyperventilating. If untreated, these events can lead to coma and death.

### Treatment

For treatment of Type 1 diabetes, insulin is essential. Human insulin is now produced by recombinant DNA technology, rather than via extraction from the pancreases of animals. Diet and exercise are key to treatment of Type 2 diabetes and this can be combined with drug treatment.

## Cardiovascular disease – atherosclerosis

### Introduction

Atherosclerosis, also known as hardening of the arteries, is a chronic arterial disease that develops over many decades and is a major cause of deaths worldwide. A raised patch or plaque, develops in the arterial wall that is rich in fat, cholesterol and calcium, and over time this hardens and narrows the artery depriving the region supplied by the blood vessel of oxygen (ischaemia). Rupture of the plaque causes blood cell fragments called platelets to stick to the surface of the injury, leading to thrombosis (formation of a blood clot) which can result in a total blockage of the affected artery. If a coronary artery is affected, a myocardial infarction (heart attack) may result or if a cerebral artery supplying the brain is affected ischaemic stroke may result. Multiple risk factors have been identified for development of atherosclerosis. Some of these are modifiable, such as an unhealthy blood lipid profile, high blood pressure, Type 2 diabetes, smoking, obesity, stress and physical inactivity. Other factors such as age, gender, race and a family history of heart disease cannot be changed. The biochemistry of lipid metabolism and process of atherosclerosis are discussed below.

### Cholesterol metabolism and lipoproteins

**Figure 2:**
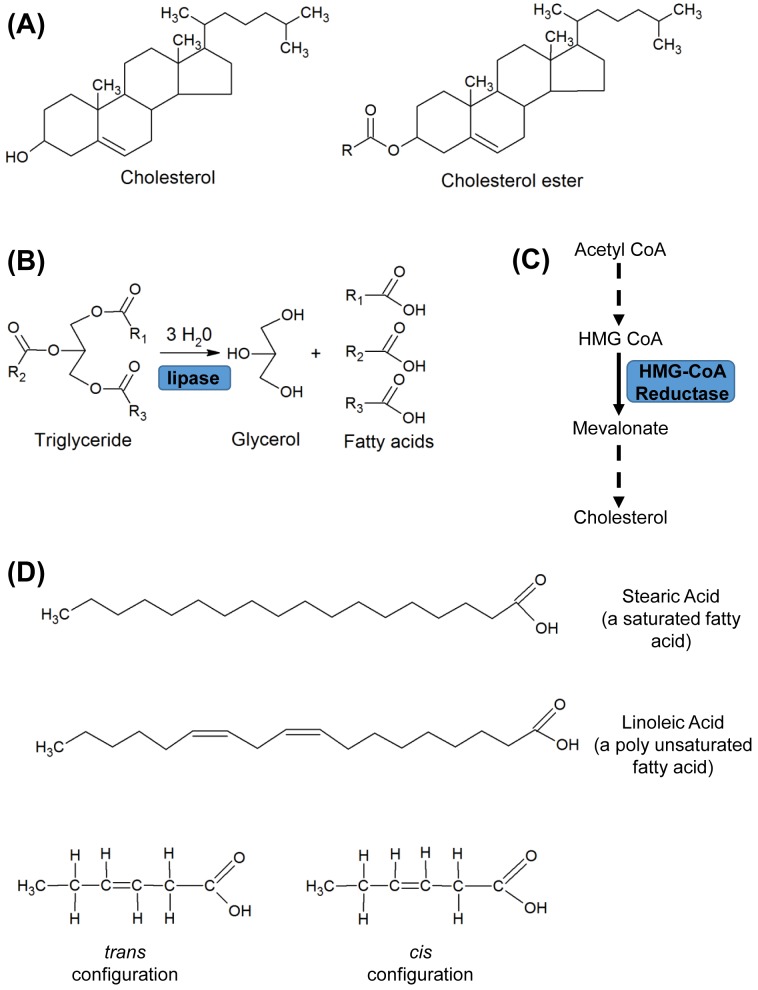
Structure and metabolic pathways for some common lipids (**A**) Structures of cholesterol and cholesterol ester. In cholesterol ester, the R group is a fatty acid as shown in (D). (**B**) Hydrolysis of triglyceride to glycerol and fatty acids by a lipase. There are several different lipases (e.g. lipoprotein lipase of endothelial cells and hormone-sensitive lipase in adipocytes). **(C)** Key steps in the multistep synthetic pathway of cholesterol. HMG CoA, 3-hydroxy-3-methylglutaryl-CoA. HMG CoA reductase is the rate-limiting step. (**D**) Fatty acids are carbon chains (most commonly 12–22 carbons) with a methyl group at one end and a carboxyl group at the other. Saturated fatty acids are ‘filled’ (saturated) with hydrogen and have no double bonds. Monounsaturated fatty acids (MUFAs) have one carbon–carbon double bond which can occur in different positions. These MUFAs may have a double bond with hydrogens in the *cis* configuration (i.e. hydrogens at either side of the double bond are orientated in the same direction) or the *trans* configuration (i.e. hydrogens are orientated in different orientations). The *cis* configuration introduces a kink in the molecular shape of the carbon chain altering physical properties. Polyunsaturated fatty acids (PUFAs) have more than one double bond. The letter *n* or Greek symbol ω, is used to indicate the position of the bond closest to the methyl end. For example, n−6 PUFAs are characterised by the presence of at least two double bonds with the first between the sixth and seventh carbon from the methyl end.

Cholesterol and fatty acids are two common types of lipids, defined as water-insoluble molecules in cells, that are soluble in organic solvents (Figure 2). Both molecules have important biological functions. Cholesterol is an important component of cell membranes where it modulates fluidity, and a precursor of vitamin D and steroid hormones produced by the adrenal gland, testes and ovaries. It is also used as a starting point for the synthesis of bile acids in the liver, which are secreted into the intestine where they solubilise fats and aid in the absorption of fat-soluble vitamins (A, D, E and K). Fatty acids are precursors of membrane phospholipids and glycolipids, and are fuel molecules that are stored as triglycerides (esters of glycerol and three fatty acids) (Figure 2).

Since lipids are insoluble in water, they are transported in the plasma as protein–lipid complexes (lipoproteins), which are divided into different types (chylomicrons, very low-density lipoproteins (VLDL), low-density lipoproteins (LDL), high-density lipoproteins (HDL)) based on their size, lipid composition and the type of protein they contain. The proteins embedded in the lipoproteins have a stabilising function and are recognised by specific receptors in the liver and peripheral tissues. In the exogenous pathway, dietary fat in the small intestine is dispersed into small droplets by bile acids and broken down into fatty acids and glycerol. Once in the enterocyte (cell lining the small intestine), the fatty acids are synthesised into triglycerides again, and packaged into lipoproteins called chylomicrons together with a small amount of absorbed cholesterol, which has been converted into its ester form. Each chylomicron contains several different apoproteins (apoB-48, apoA-I, apoA-II) and acquires apoC-II and apoE. The chylomicrons pass via the lymphatic system and blood capillaries to muscle and adipose tissue. Here the enzyme lipoprotein lipase, on the surface of endothelial cells, breaks down most of the triglycerides into glycerol and fatty acids. These molecules are taken up by the peripheral tissues and either used as an energy source or stored. The remnant chylomicrons which are depleted in triglycerides but still contain the bulk of their cholesterol ester pass to the liver and, following binding of apoE to the LDL receptor on hepatocytes, the entire particle undergoes endocytosis, resulting in cholesterol being taken up by the liver. From here the cholesterol may be stored, converted into bile acids, secreted directly in bile or may enter the endogenous pathway.

In the endogenous pathway, the liver produces VLDL particles with newly synthesised triglyceride and a small amount of cholesterol ester. These particles deliver glycerol and fatty acids to peripheral tissues, as described above for chylomicrons. Removal of the triglyceride fraction from the particles, while retaining the cholesterol component, results in their conversion into intermediate density particles and ultimately LDL particles, laden with cholesterol ester. These LDL particles are the main carrier of cholesterol to cells for incorporation into membranes and steroid synthesis, but also play a key role in development of atherosclerosis by depositing lipid in the wall of blood vessels. The surface of the LDL particle contains apoB-100 which is a ligand (i.e. binds) for the LDL receptor located on pits on the surface of the hepatocyte. Apo-B-100 binding to the LDL receptor results in internalisation of the particle and its removal from plasma. The cholesterol content of the liver cells in turn regulates the levels of LDL receptors and other key genes involved in cholesterol and fatty acid metabolism in order to maintain a balance. The genes that are regulated include the enzyme HMG CoA reductase which is the rate-limiting enzyme in the multistep cholesterol synthesis pathway (Figure 2). The levels of LDL receptor are also regulated by the secreted proprotein convertase subtilisin/kexin type 9 (PCSK9) which binds to the receptor and enhances its degradation in lysosomes. Cholesterol can return to plasma from tissues in HDL particles. HDL particles take up cholesterol, converting it into its ester form in the process, and from here it is transported away from the periphery to the liver. This may occur indirectly via transfer to VLDL particles or directly by a process involving the scavenger receptor B1 in hepatocytes which selectively takes up HDL cholesterol.

### Disease process

**Figure 3:**
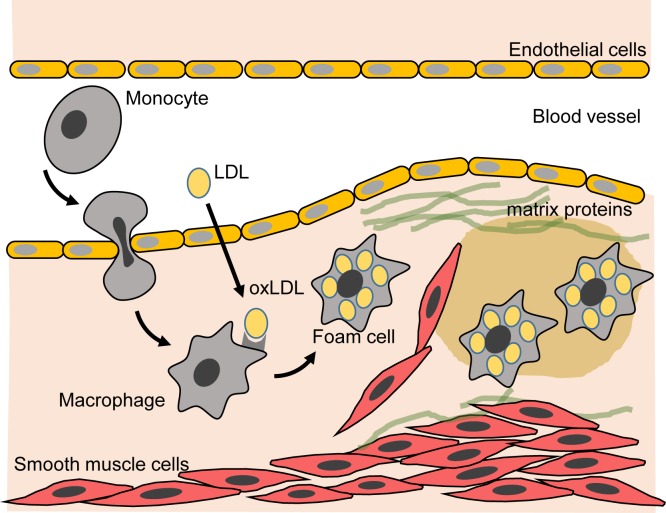
Lipoproteins and the process of atherosclerosis See text for the description of the processes involved. Adapted from Heinecke, J.W. (2006) Lipoprotein oxidation in cardiovascular disease: chief culprit or innocent bystander? *J. Exp. Med.***203**, 813–816; https://doi.org/10.1084/jem.20060218

Atherosclerosis involves damage to, or dysfunction of, the endothelial cells that form the inner lining of blood vessels, resulting in entry of LDL particles into the vessel wall (Figure 3). Lipids and proteins of the LDL particle undergo oxidation by reactive oxygen species (e.g. superoxide, O_2_^−^), generated via oxidative stress, to form oxidised LDL (oxLDL). OxLDL molecules participate in atherosclerotic plaque formation in several ways. They activate endothelial cells, promoting movement of monocytes and T cells into the vessel wall. Also the oxLDL is taken up by macrophages via ‘scavenger’ receptors resulting in conversion of the macrophages into lipid-rich foam cells. Accumulation of these cells give rise to the appearance of ‘fatty streaks’ within the endothelium. Various pro-inflammatory mediators are produced during this process which stimulate smooth muscle cell proliferation, and migration of these cells into the subendothelial layer. Matrix proteins such as collagen are deposited in large quantities by the smooth muscle cells leading to formation of a dense fibrous cap overlying the lipid-rich core. The plaque may partially block the lumen of the blood vessel or eventually rupture leading to formation of a thrombus as blood platelets adhere to the exposed subendothelial collagen.

### Risk factors

Population studies have identified a major role for the type and amount of dietary fat in determining serum cholesterol, and established a strong correlation between total plasma cholesterol, in particular high LDL cholesterol, and coronary heart disease. While high LDL cholesterol, which makes up approximately 70% of total cholesterol, is associated with disease, HDL cholesterol levels are inversely correlated with disease. One of the earliest population studies, started more than 50 years ago, revealed that plasma cholesterol and deaths from coronary heart disease were substantially lower in southern Europe and Japan, while rates in North America and northern Europe were higher. The differences were strongly associated with levels of saturated fat consumption and have led to recognition of the healthy Mediterranean diet.

It is now recognised that different types of dietary fats have distinct effects on cardiovascular disease risk and the type of fat is more important than the total amount. Current evidence indicates that replacing saturated fats with unsaturated fats (especially polyunsaturated fatty acids (PUFAs)) reduces cardiovascular disease risk. Studies of the native Inuit people living in the northern part of Greenland who have a diet rich in fish, and low coronary heart disease risk, have led to the recognition that n−3 PUFAs, such as eicosapentaenoic acid from fish, are protective against coronary heart disease. The cardiovascular benefits have been linked to anti-inflammatory effects of n−3 PUFAs, and effects on cardiac muscle cell electrophysiology and membrane fluidity. On the other hand, industrially produced *trans* fats, found in many processed foods, are associated with an increased risk of coronary heart disease. The recognition that industrially produced *trans* fats in the diet are not safe has led the U.S. Food and Drug Administration to phase out this type of fat from the food supply chain, with a deadline of 2018. Also, it is now recognised that a reduction in calories from fat, together with a compensatory increase in dietary carbohydrate from refined sugars and starches to compensate, is not a healthy approach as this is known to be associated with an increased prevalence of obesity and Type 2 diabetes.

Several genetic defects in the LDL receptor and apoprotein genes cause hyperlipidaemia and are associated with an increased risk of coronary heart disease, if untreated. Heterozygous familial hypercholesterolaemia (where one copy of the faulty gene is present) is relatively common, with 1 in 500 of the normal population affected, and is due to mutations in the LDL receptor. The mutations cause underproduction of the receptor and reduced clearance of the LDL cholesterol by the liver. The homozygous form of the disease (two copies of the faulty gene are present) is very rare and leads to highly elevated LDL cholesterol and premature death from coronary heart disease. Mutations in the apoproteins that function as ligands for the LDL receptor (e.g. apo B-100 and apoE) can cause high LDL concentrations and an increased risk of atherosclerosis.

It is worth highlighting that in addition to diet and genetics, there are many other factors that are recognised as risk factors for atherosclerosis (age, gender, smoking, high blood pressure, obesity, Type 2 diabetes, stress and physical inactivity).

### Lipid-lowering drugs

There are a number of drugs that are used clinically to lower lipid levels and reduce the risk of cardiovascular disease. Two classes of drugs of note are the ‘statins’ and recently introduced PCSK9 inhibitors. Statins, such as simvastatin and lovastatin, inhibit the rate-limiting enzyme in the multistep cholesterol synthesis pathway which converts HMG-CoA into mevalonate leading to decreased hepatic cholesterol synthesis (Figure 2C). Consequently, there is an increase in hepatic LDL receptor expression and increased clearance of LDL cholesterol from plasma into liver cells, thereby lowering plasma LDL cholesterol levels. PCSK9 inhibitors used clinically are monoclonal antibodies that lower LDL cholesterol levels by inactivating the hepatic protease (PCSK9) that attaches to and internalises LDL receptors promoting their destruction. These drugs lower plasma LDL cholesterol levels by preventing LDL receptor destruction and are useful for patients who are intolerant to statins or have severely high cholesterol levels.

Although oxLDL plays a well-established role in the process of atherosclerosis, clinical trials of antioxidant molecules, such as vitamin E, for prevention of atherosclerosis and cardiovascular disease have not demonstrated any benefit.

## Cancer

### Introduction

Cancer is characterised by unregulated cell growth, leading to invasion of the surrounding tissue and spread (metastasis) of cells to other parts of the body. The abnormal growth, or tumour, may be broadly classified as benign (i.e. grows locally without invading adjacent tissues) or malignant (i.e. invades nearby tissues and metastasises). Although the majority of tumours in humans are benign and harmless to their host, some can be life-threatening because of their location pressing on vital organs (e.g. brain tumour) or because of hormones they release (e.g. thyroid adenomas). Most cancer deaths are due to malignant tumours, specifically the metastases that arise. The World Health Organisation estimates that there were 8.8 million deaths from cancer in 2015, and cancer is one of the leading causes of mortality worldwide, with more than two-thirds of deaths occurring in the developing world. Cancers are most often described by the part of the body they originated in and more than 200 different types of cancer have been documented, many of which occur with vastly different frequencies in different population groups or geographic areas. Overall, lung, liver, stomach and breast cancer cause the most cancer deaths.

### Causes

Cancer is considered to be initiated as a result of genetic aberrations at the cellular level with biochemical and genetic evidence indicating that tumours arise from one ancestor cell (i.e. they are clonal). The causes are multifactorial, and combine individual genetic predisposition with environmental factors (Table 1). Genetic aberrations (i.e. such as single-point mutations, large chromosomal deletions, amplifications or translocations in DNA) may occur spontaneously, following a failure in cellular DNA damage repair or recognition mechanisms, during the enormous amount of cell turnover in the body throughout the course of a human lifetime (referred to as somatic mutations). Alternatively, mutations may be caused by environmental factors (chemical carcinogens, UV exposure or an infectious agent) or be due to inherited genetic factors (referred to as germline mutations). The Knudson hypothesis, formulated by Alfred Knudson in 1971, suggested that two ‘hits’ to DNA are necessary to cause cancer. This requirement for an accumulation of mutations explains the increased risk of cancer with age, as a consequence of the increased time available to acquire a mutation, and explains the documented increased cancer incidence in our population, as we live longer. The genes most commonly affected are involved in the biological processes that are recognised as the six ‘hallmarks’ of cancer: sustaining proliferative signalling; evading growth suppressors; activating invasion and metastasis; enabling replicative immortality; inducing angiogenesis and resisting cell death. More recently, this model has been updated to include several other factors.

**Table 1:** Causes of and risk factors for cancer

Genetics	Mutations associated with carcinogenesis may accumulate during DNA replication over time as we age or be inherited (germline mutations)
Smoking	Tobacco smoke contains more than 7000 chemicals, at least 60 of which cause cancer. Examples include benzene, formaldehyde and polycyclic aromatic hydrocarbons
Obesity	A high body mass index (BMI), a useful measure of obesity, is strongly correlated with an increased risk of various cancers
Alcohol	Drinking too much alcohol is well established as a cancer risk factor
Ionising radiation	X-rays and γ-rays can damage DNA directly or react with water to produce damaging intermediates (reactive oxygen species)
UV radiation	UV radiation from the sun is carcinogenic. UV-B is the most effective carcinogen and causes pyrimidine (thymine and cytosine) dimers in DNA leading to mutations
Chemicals	Many chemicals in the environment may cause cancer. Some chemicals may act directly on DNA while others are metabolised in the liver to yield the ultimate carcinogen. Many dietary components may increase or decrease cancer risk; however, with a few exceptions direct evidence demonstrating carcinogenic or protective effects in humans has not been obtained
Infectious agents	Both viruses and bacteria are recognised as causative factors in various cancers: e.g. human papilloma virus – cervical cancer, hepatitis B virus – liver cancer; *Helicobacter pylori* (*H. pylori*) – gastric cancer
Reproductive life	Breast cancer risk in women is influenced by reproductive history: e.g. not having children, age at giving birth for the first time, and hormonal contraceptive and hormonal replacement therapy

For more in-depth discussion of the vast literature on cancer biology, the reader is recommended to consult one of the many excellent textbooks on the topic (see ‘Further reading’ section). The discussion below examines examples of biochemical aspects of cancer associated with gain-of-function mutations in certain proto-oncogenes (i.e. genes that when altered by mutation contribute to cancer) and how loss-of-function of tumour suppressor genes, which normally suppress growth, can be linked to cancer.

### Chronic myeloid leukaemia

Chronic myeloid leukaemia (CML) is a rare leukaemia which starts in the bone marrow, the sponge-like tissue inside bones, where blood cell formation starts. It almost exclusively affects adults during or after middle age, and progresses slowly from a chronic phase, which can last several years, to an acute phase and blast crisis, which can be fatal. In CML, a chromosomal translocation (i.e. a swap of DNA sequences on different chromosomes) results in changes to chromosomes 9 and 22. Part of chromosome 22, at a region known as the break-point cluster region (BCR), becomes fused to the *ABL* gene from chromosome 9, creating what is referred to as the Philadelphia chromosome, named after the city of its discovery, and the BCR-ABL protein. This genetic change in the myeloid stem cells of the bone marrow, which normally develops into granulocytes (basophils, neutrophils and eosinophils), results in overproduction of abnormal cells of this type, and there is less room for formation of other blood cell types (red cells, platelets and white blood cells). As a result patients may have anaemia, weight loss, easy bleeding and abdominal pain due to an enlarged spleen.

The human *ABL* gene encodes a non-receptor tyrosine kinase. This is an enzyme which can transfer a phosphate group from ATP to the amino acid tyrosine in substrate proteins. In response to extracellular signals such as growth factors or cytokines, ABL is activated to stimulate complex cell signalling pathways involved in cell proliferation and survival. The ABL protein is composed of several functional domains (compact folded units within a protein) including the kinase domain which has catalytic activity, and normally cellular activity of ABL is low. Protein structural studies by X-ray crystallography have revealed that activity is held in check by an auto-inhibition mechanism, in which a lipid moiety (myristate) that is covalently attached to a sequence near the start of the protein (i.e. the N-terminus) loops around and is inserted into the kinase domain, to keep the enzyme in an inactive state. This auto-inhibition mechanism is lost from the BCR-ABL protein, because the important N-terminal amino acid sequence in ABL is replaced by a sequence from the *BCR* gene, resulting in a constitutively active (i.e. constantly active) form of the kinase that causes cellular changes leading to leukaemia.

A number of inhibitors of the BCR-ABL tyrosine kinase have been developed which are highly useful clinically for treating this leukaemia, the first of which was Imatinib (Gleevec). This successful therapeutic approach, which is often regarded as the first targeted cancer therapy, has given rise to the development of many other kinase inhibitors for other cancers (e.g. breast cancer, melanoma) and inflammatory diseases (e.g. rheumatoid arthritis).

### Epidermal growth factor receptor and related family members

**Figure 4:**
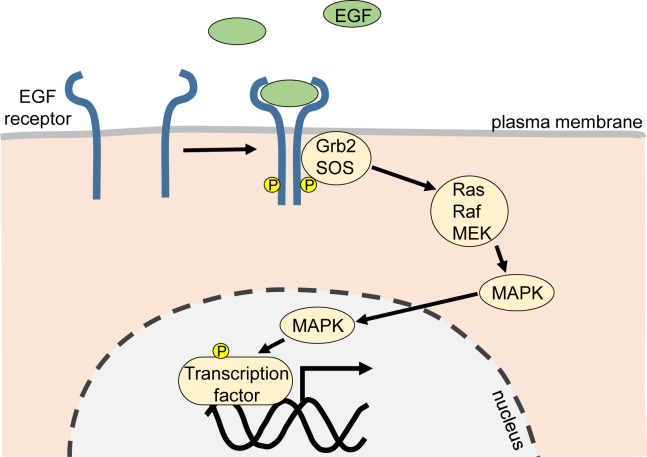
The signal transduction pathway of the EGF receptor MAPK affects the activity of transcription factors via phosphorylation. Abbreviations: Grb2, growth factor receptor-bound protein 2; MAPK, mitogen-activated protein kinase; MEK, a mitogen activated protein kinase kinase; Raf, a serine/threonine protein kinase activated by Ras; Ras, a small GTPase protein; Sos, Son of Sevenless (a nucleotide exchange factor).

The biochemistry of the epidermal growth factor (EGF) receptor and related family members, provides a useful example of how a cell surface protein can respond to an extracellular biomolecule signal and convey that message to the interior of a cell to regulate cell proliferation or invasion. This pathway is of particular relevance to a discussion of cancer, since it is known that a substantial number of tumours carry gene amplifications that lead to elevated EGF receptor levels, or deletions or point mutations. The EGF family of receptors consists of four closely related receptor tyrosine kinases: ErbB1 (EGF-R, HER1), ErbB2 (HER2, Neu), ErbB3 (HER3) and ErbB4 (HER4). The receptors are activated following binding of a ligand (EGF or other ligands) and dimerisation. Dimerisation refers to the process whereby receptor proteins pair up with one another to form homodimers (i.e. a receptor pair formed of the same type of receptors) or heterodimers (i.e. a receptor pair formed of different receptors). Variations to this process are found with HER2 which has no known ligand and HER3 lacks kinase activity, but both have important cell signalling functions via the heterodimers they form. Following dimerisation, the close proximity of the two receptor molecules allows the kinase of one molecule of the pair to phosphorylate the other on specific tyrosine amino acids (a process referred to as transphosphorylation). Subsequently, signalling proteins associate with the phosphorylated receptor initiate a cascade of signalling events culminating in activation of transcription factors in the nucleus and changes in gene expression regulating cell growth and proliferation (Figure 4). The pathway is tightly regulated by processes that ‘switch off’ signalling, such as phosphatases (enzymes that cleave phosphate from their substrate), and degradation of the receptor.

The *HER2* gene is amplified in approximately 30% of human breast cancers. The resulting overexpression of this protein, often at levels 10–100-times above normal, can drive spontaneous dimerisation via mass-action effects, and activation of cell signalling pathways linked to growth, division and protection from programmed cell death (apoptosis), to stimulate the malignant phenotype. Other mutations or truncations in EGF receptor family proteins can cause ligand-independent activation of the receptor. A variety of clinically useful monoclonal antibodies, and small molecule kinase inhibitors, have been developed against EGF receptor family proteins with the intent of treating tumours that exhibit high-level expression of the receptors. The monoclonal antibody trastuzumab (Herceptin) is an anti-HER2 antibody that has resulted in an extension of lifespan for breast cancer patients, and has been approved for treatment of gastric carcinomas that overexpress HER2. Its precise mechanism of action is not entirely clear but it is thought to involve ‘tagging’ HER2 expressing cells and essentially marking them for elimination by cytotoxic cells of the immune system.

### Tumour suppressor p53

Growth promoting genes, such as those discussed above, represent only part of the story of cellular growth control, with the other part consisting of genes that suppress uncontrolled growth and are called tumour suppressor genes. There are many genes in this category (e.g. *RB1, BRCA1, BRCA2, PTEN*); however, the *TP53* gene and its product, the p53 protein, plays such a key role as a tumour suppressor it is often referred to as ‘the guardian of the genome’. Studies of cancer cell genomes from a wide range of tumours indicate that p53 is the gene found to be most frequently mutated. In normal healthy cells the levels of p53 are low but expression is increased in response to cell stresses such as radiation, certain chemotherapeutic drugs, DNA damage, low oxygen tension (hypoxia) and oncogene signalling. The p53 protein is a transcription factor and activates genes involved in: arrest of the cell cycle (i.e. the series of events that regulate cell division and DNA replication); DNA repair; blocking angiogenesis (i.e. blood vessel formation) and apoptosis (i.e. programmed cell death). Overall, when cells detect damage or abnormal functioning, they send signals to p53 which acts by halting cell proliferation or triggering apoptosis. Thus the absence of p53 in a tumour cell will permit the survival of cells that are accumulating mutations and allow tumour development.

A few of the many important genes that are induced by p53 to exert tumour suppressing activities are p21, Bcl-2 related genes, XPC and TSP-1 (thrombospondin). Induction of the *p21* gene inhibits cyclin-dependent kinases, which are involved in the cycle process, resulting in arrest of the cell cycle at the first checkpoint (i.e. transition from G_1_ preparation for DNA synthesis to S phase DNA synthesis). This allows the cell to repair DNA damage. If successful, the cell proceeds into S phase; if not, apoptosis pathways are activated. Pro-apoptotic members of the Bcl-2 family of genes are induced by p53 and these outcompete anti-apoptotic family members. The intracellular site of action of these proteins is the mitochondria and apoptosis is triggered by opening of pores in the mitochondrial membrane, allowing the contents to spill out. Induction of the *XPC* gene by p53 increases the cell’s ability to locate and repair DNA damage, while induction of the *thrombospondin* gene, an inhibitor of new blood vessel formation, prevents cancerous cells from developing a blood supply during early tumour development.

### How many types of cancer?

More than 200 different types of cancer have been documented based on their cell type of origin in the body (as above); however, defining distinct diseases is complex as recent studies have shown. Analysis of the genetic profile of more than 1500 patients with the blood cancer, AML indicated that they could be grouped into 11 distinct classes each with specific diagnostic features and clinical outcomes. On the other hand, analysis of 11000 tumours from 33 of the most prevalent forms of cancer, by The Cancer Genome Atlas (TCGA) consortium, has revealed that cancers with different tissue or cell origins are genetically similar. These findings may provide the basis for new therapeutic strategies.

## Microorganisms

### Cholera

Cholera is an acute diarrhoeal illness that kills approximately 100000 people worldwide each year. The World Health Organisation reported in 2018 that the outbreak of cholera in Yemen is the largest and fastest spreading outbreak of the disease in modern history, with more than a million people affected. The disease is caused by the bacterium *Vibrio cholerae* and spread by consuming contaminated water and food polluted with sewage (the faeco-oral route). It typically affects regions where there is overcrowded housing and water and sanitation are poor, or where conflict or a natural disaster have led to collapse of the water, sanitation and the healthcare systems. In 1854 the physician John Snow traced an outbreak of cholera in London to a water pump in Soho, which was taking sewage-polluted water from the Thames, and established the water-borne nature of the disease.

**Figure 5:**
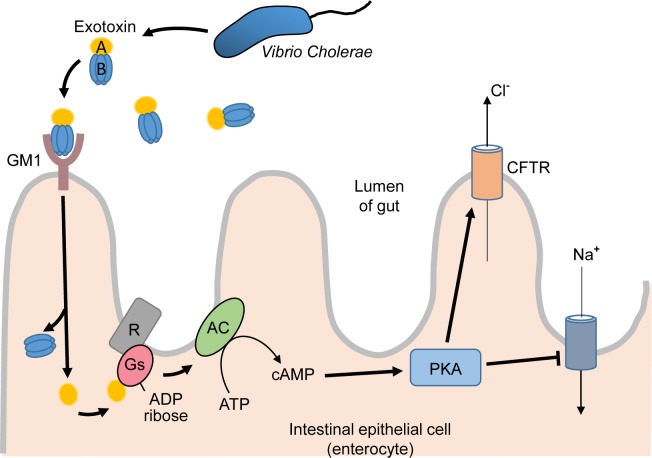
The pathogenesis of cholera Cholera exotoxin (a toxin released by the bacterium *V. cholerae*). See text for a description of the process. Abbreviations: AC, adenylate cyclase; R, G-protein-coupled receptor.

In the small intestine of affected individuals, *V. cholerae* secretes a toxin (referred to as exotoxin) consisting of an active A subunit attached to a ring of five B subunits. The B subunits bind to a cell surface receptor (ganglioside receptor GM1 (GM1)) on the epithelial cells lining the gut (Figure 5). The receptor–toxin complex is endocytosed and transported to the endoplasmic reticulum where the A subunit dissociates from the B subunit to enter the cytosol. The A subunit has enzymatic activity and transfers ADP-ribose from NAD^+^ to a protein guanine nucleotide-binding protein (or G protein) called Gs (stimulatory G protein), that is a part of the signalling pathway in mammalian cells that some hormones use. Normally in this pathway, a hormone binds to a G-protein-coupled receptor which activates the G protein (composed of three different subunits: α, β and γ) causing exchange of GDP for GTP on the α subunit. The GTP-bound α subunit then activates the enzyme adenylate cyclase leading to production of cAMP. The cycle is switched off by the G protein α subunit itself which has a built-in enzymatic GTPase activity (i.e. it converts GTP into GDP). The cholera toxin ADP-ribosylation of the Gs α subunit irreversibly inhibits the intrinsic GTPase of the Gs, locking it in the active state, leading to a sustained activation of adenylate cyclase and a dramatic increase in cAMP levels within the cell. The cAMP activates cAMP-dependent protein kinase (protein kinase A, PKA) which phosphorylates and stimulates the cystic fibrosis transmembrane conductance regulator (CFTR), a channel protein in the plasma membrane, leading to changes in the electrolyte balance across the cell membrane. There is an increase in chloride and bicarbonate movement out of the cell, a decrease in sodium influx and a corresponding movement of water molecules into the lumen of the gut, and net fluid loss causing watery diarrhoea. It is interesting to note that another bacterial toxin (pertussis toxin causative factor of Whooping cough) functions by a similar mechanism, albeit with different cell types affected.

Treatment for cholera is relatively cheap and simple. A simple rehydration solution prepared with boiled or bottled water is used to replace lost fluids and electrolytes. In severe cases, fluid via the intravenous route may also be required. In addition, cholera vaccines are available which offer some degree of protection; antibiotics may also be used in severe cases to reduce disease duration. It is interesting to note that the cystic fibrosis gene, in which there is dysfunction of the CFTR leading to production of thick mucus, may have survived evolutionary pressure because it gives resistance to cholera.

### HIV

HIV, is the virus that causes AIDS. It results in a profound weakening of the immune system leaving patients vulnerable to other infections and complications. Since its first description in the early 1980s, it has claimed more than 35 million lives and more than 37 million people are living with HIV around the world. There is currently no cure but effective antiretroviral drugs can control the virus and prevent transmission. Wider access to these drugs and HIV prevention programmes have reduced HIV-related deaths and new infections to their lowest point in over two decades. Here, biochemical aspects of how the HIV virus penetrates a living host cell and uses the host’s metabolic machinery to replicate are discussed.

**Figure 6:**
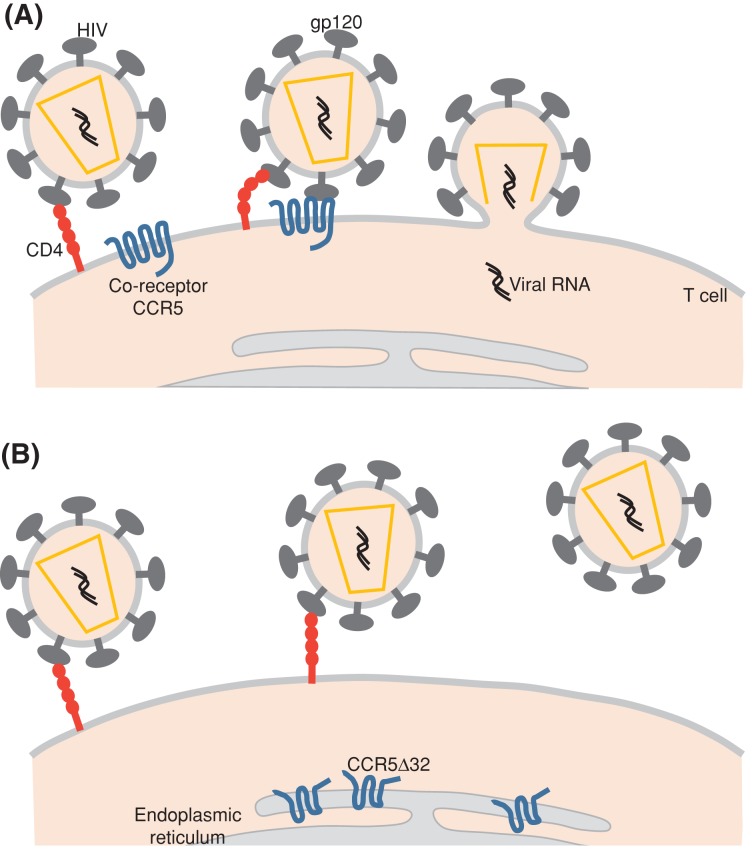
HIV entry via cell surface receptors (**A**) CCR5 serves as a co-receptor with CD4 to permit HIV entry. (**B**) The CCR5Δ32 variant produces a mutant protein that does not reach the cell surface and is non-functional as a co-receptor. See text for details.

HIV is a retrovirus (i.e. it contains a reverse transcriptase enzyme that can synthesise DNA from viral RNA). Two forms of the virus, HIV-1 and HIV-2, are known and both cause immunosuppression but it is the HIV-1 strain that is most frequently occurring and virulent. The virus infects cells of the host’s immune system, specifically CD4^+^ helper T cells lymphocytes, macrophages and dendritic cells that are normally involved in co-ordinating the immune response to a disease-causing organism. CD4 is a protein found on the surface of immune cells, and a viral envelope glycoprotein, termed as gp120, binds to CD4 to gain entry into cells. It cannot do this alone and an additional co-receptor is required for entry. One such co-receptor is a G-protein-coupled receptor named chemokine receptor type 5 (CCR5) that is normally a receptor for specific chemokines (i.e. small secreted protein molecules that play a role in directed movement of cells), namely MIP-1 and RANTES. Some strains of the virus are able to use a different chemokine receptor (CXCR4) together with CD4 for entry into cells (Figure 6). HIV is not unique in its ability to exploit normal membrane receptors as a means to gain entry into cells and in fact a long list of viruses (e.g. rhinovirus, hepatitis C virus) use a variety of cell surface receptors to enter cells.

The importance of CCR5 as a co-receptor *in vivo* has been demonstrated by the discovery of a genetic variant of the *CCR5* gene, found in approximately 10% of Caucasians, that confers resistance to HIV infection. The CCR5 receptor, as with other members of the G-protein-coupled receptor family, is characterised by seven transmembrane spanning domains with the N-terminus outside the cell and the C-terminus inside the cell. The CCR5Δ32 variant of the gene contains a 32-bp deletion within the second extracellular loop that produces a frameshift mutation and premature stop codon, and consequently the mutant protein does not reach the cell surface and is retained within the cell in the endoplasmic reticulum, where it is non-functional, either as a chemokine receptor or HIV co-receptor. Individuals who are homozygous (i.e. have two copies) of the Δ32 variant are resistant to HIV infection, although may be susceptible to strains of the virus using a different co-receptor. A drug for treating HIV infection, maraviroc, which binds to the CCR5 receptor and blocks virus entry is used clinically.

Once inside the cell, the viral RNA is copied into double-stranded DNA by the viral enzyme reverse transcriptase. This is an error-prone enzyme resulting in the introduction of a large number of mutations into the viral genome, which leads to its ability to evade the human immune system. The virus-specific reverse transcriptase enzyme is a useful drug target for several important antiviral nucleotide analogues. These drugs are modified by the cell, and incorporated into the viral genome and ultimately block elongation of the DNA chain. In untreated cells, viral DNA is incorporated into the host DNA and subsequently transcribed and translated to form new virus particles that are released from the cell and initiate another round of infection.

## Nutrition

### Introduction

Food is necessary to provide the body with energy and key biomolecules that are essential for normal body function. Disease may be associated with an excess intake of energy-rich foods, undernourishment or malnutrition. The components of food that are digested and absorbed by the body can be divided into macronutrients (carbohydrates, fats and proteins) that provide energy and micronutrients (vitamins and minerals) which do not provide energy, but are required in small amounts. An overview of biochemical aspects of nutrition is provided here, together with a focus on selected current issues and areas of interest.

### The biochemical nature of macronutrients and their functions

The group of carbohydrate molecules includes sugars, starch and fibre. They can exist as monosaccharides (such as glucose and fructose), disaccharides (such as sucrose and lactose) and polysaccharides (such as starch, glycogen and cellulose). A disaccharide is formed from two monosaccharides linked together: one glucose and one fructose molecule in the case of sucrose (table sugar), and one glucose and one galactose molecule in the case of lactose (found in milk). Polysaccharides are composed of long chains of hundreds to thousands of monosaccharides in either a linear or highly branched structure. Starch, formed from a large number of glucose units, the most common form of carbohydrate in the human diet is derived from plants and is found in potatoes and cereals. Glycogen is a highly branched polymer of glucose that serves as an energy store in humans, mainly in liver and skeletal muscle, that can be quickly broken down to supply a need for glucose. Cellulose, a polysaccharide also formed from glucose units, is a structural component of the plant cell wall and is a component of dietary fibre. Although humans are unable to digest cellulose because of a lack of the appropriate enzymes to break the β-glycosidic bonds between the glucose units (α-glycosidic bonds are found in glycogen and starch), dietary fibre is important for healthy functioning of the digestive tract.

Dietary fat is mainly in the form of triglycerides, which are made up of three fatty acid molecules linked with one molecule of glycerol (Figure 2). These fatty acids may vary in chain length, the presence or absence of double bonds within the chain (saturation) and the configuration of hydrogens at either side of the double bonds (*cis* or *trans*). The body can synthesise most fatty acids from carbohydrate or other fatty acids; however, two types of fatty acids (linoleic and α-linolenic) cannot be synthesised and a dietary source is required. These essential fatty acids are used in the synthesis of prostaglandins. Fat is stored in adipocytes (fat cells) within adipose tissue which is concentrated into characteristic areas of the body (such as beneath the skin and abdominal areas), and is an important energy source during prolonged exercise. Another molecule of note at this point is cholesterol, since both cholesterol and fats are categorised as lipid molecules (due to the fact they are water insoluble). Cholesterol is not used as an energy source but is an important component of cell membranes and as a precursor of hormones and bile salts (as discussed above). It may be obtained from dietary sources but is also synthesised by many cell types.

Proteins are a diverse group of biomolecules formed from a chain of amino acids. Dietary protein sources are meat, fish, eggs, nuts, dairy products and legumes (plants of the pea family). The thousands of proteins within the body have numerous biological functions and diverse structures. The majority of amino acids are obtained from digestion of dietary proteins; however, there are nine essential amino acids that cannot be synthesised by the body, and thus must be supplied in the diet. These are phenylalanine, threonine, tryptophan, methionine, leucine, isoleucine, lysine, valine and histidine.

Energy is required by every cell in the body, and it is through the metabolism of glucose, fatty acids and amino acids that ATP, the energy storage molecule, is generated. When required, glycogen is broken down to glucose-1-phosphate and subsequently converted into pyruvate by glycolysis. Pyruvate is then transported into the cytosol of mitochondria and converted into acetyl CoA releasing CO_2_ and water in the process. Fatty acids and amino acids can also be converted into acetyl CoA. Acetyl CoA is the starting point for the citric acid cycle (also known as the tricarboxylic acid cycle or Krebs cycle), a series of chemical reactions which generate ATP and high-energy electrons that are quickly passed to the respiratory chain in the mitochondrial inner membrane. Here the last series of reactions, in a process termed as oxidative phosphorylation, generates more ATP as a supply of energy for the cell.

### Disorders associated with macronutrients

Metabolic disorders associated with macronutrients may be linked to an excess intake of energy-rich foods, undernourishment, malnutrition, genetic errors in metabolic enzymes or adverse reactions to particular foods. The World Health Organisation have reported the massive scale of the problem of malnutrition in all its forms: 1.9 billion adults overweight or obese, 462 million underweight; 52 million children under 5 years of age are wasted (i.e. low weight for height) and 155 million have stunted growth. To tackle this problem, the United Nations declared a ‘Decade of Action on Nutrition’ from 2016–2025.

In many developing areas of the world, people are affected by malnutrition as a result of poverty, war or drought hindering access to the food supply. Severe protein-energy malnutrition has two forms: kwashiorkor and marasmus. Kwashiorkor typically occurs in a young child after a mother weans the child from breast milk and is often associated with an infection such as measles or diarrhoea. Weaning from breast milk causes a dietary change from a diet containing proteins, amino acids and fats to one consisting mainly carbohydrates. The symptoms of the disease are fluid build-up in tissues (oedema) leading to swelling of legs and ankles, dry skin rash, weakness and reddish orange discolouration of the hair. A characteristic symptom is a ‘pot belly’ or distended abdomen, as a result of abnormal fatty enlargement of the liver, and fluid build-up. Fluid build-up in tissues is due to deficient serum albumin plasma protein synthesis. A hydrostatic pressure gradient in capillaries pushes water into the tissues, and this fluid would normally be drawn back into the capillary by the osmotic pressure exerted by albumin. In kwashiorkor, low serum albumin leads to reduction in this effect leading to fluid build-up in tissues. The disorder can be treated by the gradual reintroduction of milk-based or specially formulated food products, but if untreated it is fatal.

Marasmus is a severe form of malnutrition in which there is inadequate caloric intake in all forms, including protein; in contrast with kwashiorkor where there is protein deficiency with adequate energy intake. It mostly commonly occurs in children but can affect adults. The condition is characterised by muscle wasting and loss of body fat, without the oedema of kwashiorkor, and is often accompanied by infections. Treatment is by gradual reintroduction of a balanced diet and the prognosis is better than for kwashiorkor.

**Figure 7:**
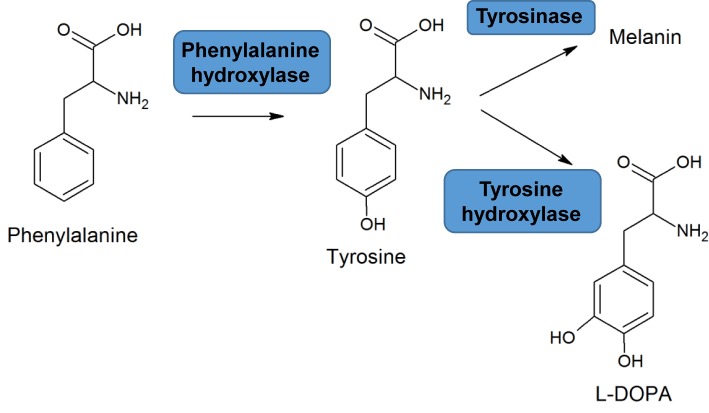
Hydroxylation of phenylalanine to tyrosine A deficiency in the enzyme phenylalanine hydroxylase leads to PKU. Melanin is a natural skin pigment. l-DOPA is a precursor of the neurotransmitters dopamine, adrenaline and noradrenaline.

There is a two-way relationship between nutrition and the human genome that determines disease risk. Just as diet can be a factor in disease for some individuals, genetic variation can lead to nutrition-linked disease, and in many cases nutrients can be considered ‘signalling molecules’ transmitting changes in gene, protein and metabolite expression that are associated with disease. Some of these relationships are discussed in the sections on atherosclerosis, obesity, alcoholic-liver disease and cancer. The conditions phenylketonuria (PKU) and lactose intolerance are examples of nutrient–gene interactions causing disease. PKU is a rare inherited disorder (affecting 1 in 10000 individuals) in which there is a mutation in the phenylalanine hydroxylase gene. This enzyme normally converts phenylalanine into tyrosine and mutations may lead to severely reduced levels of the enzyme, its complete absence or reduced enzyme activity leading to accumulation of phenylalanine, when untreated (Figure 7). Untreated PKU can lead to mental retardation, behavioural problems, epilepsy, light skin pigmentation and jerking movements of arms and legs. The light skin colour is due to deficient melanin production, resulting from lower tyrosine levels. The condition can be diagnosed in newborns by a routine blood spot test and treatment consists of a low-protein diet with amino acid supplements. Another example of a single gene defect underlying a nutrition-related disease is that of lactose intolerance. The enzyme lactase produced by the mucosal cells of the gut breaks down the disaccharide lactose of dairy products to its monosaccharides (glucose and galactose), which are absorbed in the small intestine. In some individuals, a deficiency of this enzyme means that undigested lactose passes to the colon where it is digested by bacteria producing gas and other symptoms such as diarrhoea, flatulence and cramps.

There are many other situations where dietary components or nutrients are associated with disease; where there is gastrointestinal disease that affects absorption of nutrients or where an eating disorder with a psychological basis affects nutrient intake. For example: peanut allergy, alcoholic liver disease, gastric ulcers and inflammatory conditions such as ulcerative colitis and inflammatory bowel disease and anorexia nervosa; however, coverage of these conditions is beyond the scope of this article. Coeliac disease is an interesting example of an inflammatory disease of the gastrointestinal tract involving dietary proteins, genetic factors and the immune system. It affects approximately 1% of the population and in genetically susceptible individuals it is triggered by the ingestion of proline- and glutamine-rich proteins that are found in wheat, rye and barley, and are termed as ‘glutens’. In wheat, the specific proteins are glutenins and gliadins. Because of its high proline content the gluten is only partially digested to a number of large gluten peptides. These peptides cross the epithelial mucosa barrier of the gut to the underlying lamina propria where some are modified by a tissue transglutaminase enzyme. Presentation of these peptides by specific HLA molecules (human leucocyte antigens are cell-surface protein responsible for regulating the immune response) on antigen presenting cells activates T cells to produce inflammatory cytokines, particularly interferon γ. This starts an inflammatory cascade which leads to a loss of the villi on the surface of the small intestine resulting in a flattened surface characteristic of the disease, which has a reduced capacity to absorb nutrients. Most individuals respond to treatment with a ‘gluten-free’ diet.

### Overview of micronutrients

The term micronutrient encompasses vitamins and minerals that are required in small amounts by the body. Essential vitamins cannot be synthesised by the body and must be obtained from dietary sources. However, a few vitamins are produced by bacteria in the gastrointestinal tract (e.g. Vitamin K) and others can be synthesised from precursor molecules (e.g*.* Vitamin D from 7-dehydrocholesterol; niacin from tryptophan; vitamin A from β-carotene). The 13 recognised vitamins can be divided into biomolecules that are fat-soluble and those that are water-soluble. The fat-soluble vitamins are often stored in adipose tissue, while there is no storage of most water-soluble vitamins and a daily dietary supply is required. Vitamins have diverse roles as hormones (vitamin D), signalling molecules (vitamin A) and as coenzymes, combining with enzymes to facilitate a range of biochemical enzymatic processes (Table 2). A coenzyme that is tightly or covalently bound to an enzyme is termed a prosthetic group. These coenzymes often function in the mechanism of catalysis by acting as donors of specific chemical groups or electrons.

**Table 2:** Vitamins and associated deficiency diseases

Vitamin	Metabolic role and coenzyme function	Deficiency disease
Fat-soluble		
A (Retinol)	Vision; cell proliferation and division; glycoprotein synthesis	Night blindness; xerophthalamia
D (Cholecalciferol)	Bone growth calcium homoeostasis; immune regulation	Rickets (children) and osteomalacia (adults) – defective bone development, bones are soft and weak
E (Tocopherol)	Protection from reactive oxygen species	Haemolytic anaemia
K (Phylloquinone)	Cofactor for γ-glutamyl carboxylase. Synthesis of coagulation factors	Coagulation defect – excessive bleeding
Water-soluble		
B1 (Thiamine)	Carbohydrate metabolism	Beriberi – accumulation of lactate and pyruvate causes peripheral vasodilation, oedema and heart failure
B2 (Riboflavin)	Role in redox reactions	Eye and skin inflammation disorders (particularly at corners of mouth)
B3 (Niacin)	Role in enzyme hydrogen donors/acceptors in redox reactions involved in oxidative phosphorylation and fatty acid synthesis	Pellagra (rare) – dermatitis, dementia and diarrhoea
B5 (Pantothenic acid)	Part of coenzyme A and acyl carrier protein (ACP) – role in citric acid cycle and lipid synthesis	N/A
B6 (Pyridoxine)	Amino acid metabolism: coenzyme pyridoxal 5′-phosphate. Also associated with glycogen phosphorylase	Weakness, peripheral neuropathy, Dermatitis
B7 (Biotin)	Coenzyme for carboxylase enzymes involved in fatty acid, amino acid metabolism and citric acid cycle	Dermatitis
B9 (Folic acid)	Tetrahydrofolate plays a role in one carbon transfer reactions in DNA synthesis	Megaloblastic anaemia and neural tube defects in pregnancy
B12 (Cyanocobalamin)	Methionine and proprionate metabolism: cofactor in enzymes methionine synthase and methylmalonyl-coA mutase	Megaloblastic anaemia and neurological dysfunction
C (ascorbic acid)	Antioxidant. Required by hydroxylase enzymes for collagen synthesis – conversion of proline into hydroxyproline. Protein metabolism	Scurvy – poor wound healing, haemorrhage, swollen gums, weakness, ‘corkscrew’ hair

Each vitamin’s name often refers to several related compounds (e.g. Vitamin A refers to retinol, retinal, retinoic acid). Only one biomolecule is named here for simplicity. Dietary sources of vitamins and values for the recommended daily intake are widely available in other texts (see ‘Further reading’ section).

Deficiency of a micronutrient leading to disease may be a consequence of inadequate dietary intake, insufficient absorption from the gastrointestinal tract as a result of disease, liver disease, lack of exposure to sunlight or changes to the gut microflora due to antibiotic use. Deficiency diseases are summarised in Table 2.

Minerals are inorganic nutrients and the daily requirement ranges from grams to micrograms. The major minerals are sodium, calcium, chloride, potassium, magnesium and phosphorus, while others such as iron, iodine, cobalt, copper, manganese, molybdenum, manganese and zinc are referred to as trace elements. They are involved in diverse functions in the body, some of which are given in Table 3.

**Table 3:** Minerals as nutrients and associated deficiency diseases

Mineral	Function	Disease (deficiency/excess)
Calcium	Component of bone together with phosphorus. Muscle contraction, enzyme activation, secretory processes, cell signalling, blood clotting	Deficiency often associated with insufficient vitamin D – weak bones
Sodium	Plasma and cellular electrolyte balance. Electrochemical activity for nerve and muscle function	Deficiency – weakness, muscle cramps; Excess – raised blood pressure
Magnesium	Cofactor for many enzymes (especially those requiring ATP). Role in bone, muscle and DNA replication	Deficiency – muscle weakness and cardiac arrhythmias
Chloride	Maintenance of body’s acid-base balance and hydrochloric acid production in the stomach	
Potassium	Main intracellular cation – muscle and nerve function	Deficiency/excess – arrhythmias
Phosphorus	Component of bone, energy transfer (ATP), component of ATP	Deficiency – weakness, osteoporosis
Iron	Component of haem in myoglobin and haemoglobin and cytochrome enzymes of electron transport system	Anaemia
Iodine	Synthesis of thyroid hormones	Deficiency – hypothyroidism lethargy, reduced metabolic rate. Effects on growth and development
Cobalt	Component of vitamin B12 (see above) red blood cell production	Anaemia
Copper	Associated with oxygenase enzymes (cytochrome oxidase and superoxide dismutase)	Anaemia

Numerous other trace metals are also required but are not listed.

### Vitamin supplements

Vitamin supplements are used by millions of individuals worldwide; however, for most healthy population groups they provide no benefit, as a healthy balanced diet provides the required vitamins and minerals (some of which may be obtained through fortification of food). On the other hand, there is good evidence that certain vitamin supplements may be beneficial for specific population groups (the elderly, pregnant mothers and children aged between 6 months and 5 years). More information is available on the NHS website and the NIH Office of Dietary Supplements website (see ‘Further reading’ section). A number of large-scale randomised trials have also shown that multivitamin use not only provides no benefit to the majority of the population but long-term use may have harmful effects.

### Vitamin D deficiency

#### Structure and synthesis

**Figure 8:**
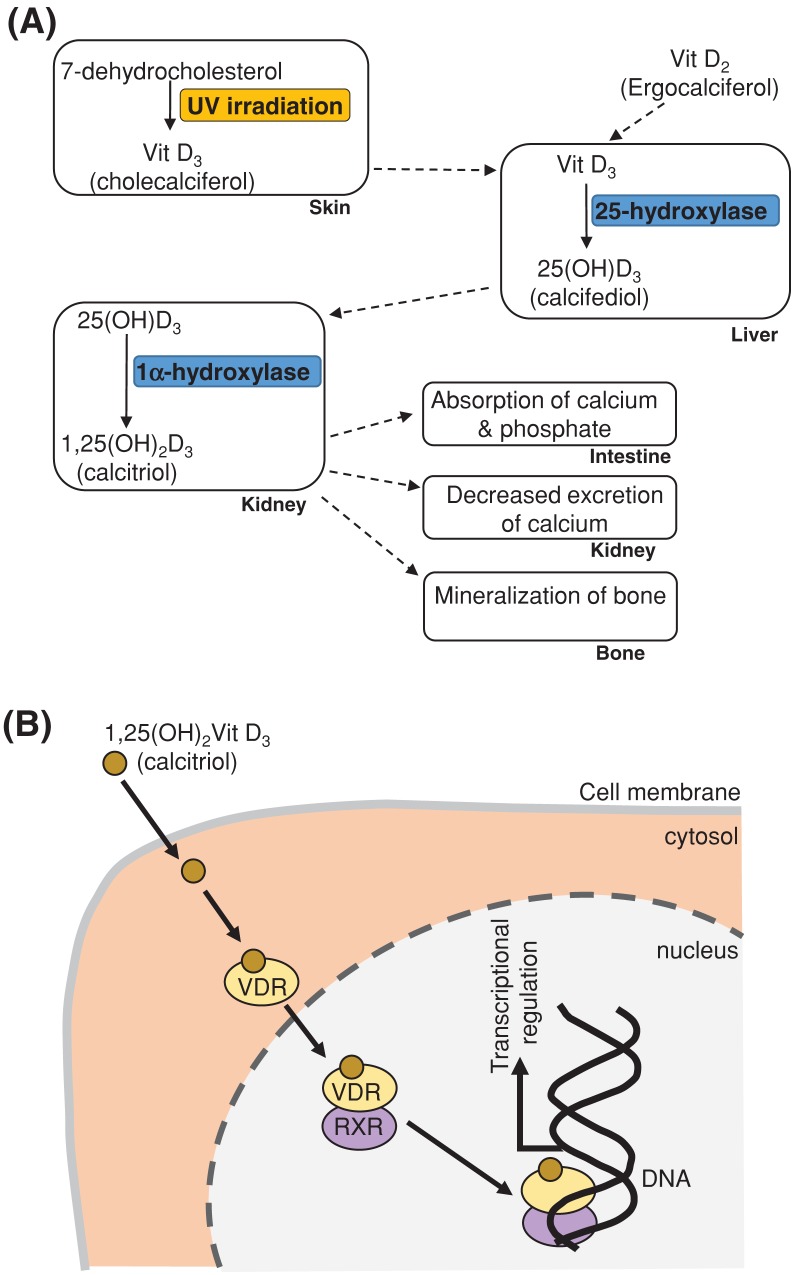
Vitamin D – synthesis, functions and molecular mechanism of action See text for details. Abbreviation: VDR, vitamin D receptor.

Vitamin D is in fact a family of steroid hormones that may be obtained from the diet or made via the actions of UV light in the skin, and it is known to play a role in regulating levels of calcium and phosphate in the body and bone mineralisation. Cholecalciferol (vitamin D_3_) is synthesised from 7-dehydrocholesterol by UV irradiation of the skin, through exposure to sunlight (Figure 8). Vitamin D_3_ can also be synthesised from ergocalciferol (vitamin D_2_), which is in turn derived from dietary ergosterol in plants. Vitamin D_3_ is converted into its most potent form 1,25 dihydroxy-vitamin D_3_ (calcitriol) by sequential enzymatic hydroxylation reactions in the liver and kidney. The 1α hydroxylation step in the kidney is stimulated by parathyroid hormone, a decrease in plasma phosphate levels and inhibited via a negative feedback mechanism involving calcitriol itself. Vitamin D_3_, and its hydroxylated metabolites, are hydrophobic molecules and are transported in the blood bound to specific globulin proteins (vitamin D-binding protein). As a fat-soluble vitamin,vitamin D is stored by the body in adipose tissue which acts as a reservoir for periods when dietary intake or UV-synthesised sources are insufficient.

#### Functions

The main actions of calcitriol are to stimulate intestinal calcium and phosphate uptake, increase bone calcium resorption and reduce loss of calcium by the kidney. These are not the only effects of calcitriol and it is now recognised that the hormone has many other important effects in many cell types including some anti-inflammatory and immune-modulating properties.

The bones of the human skeleton are continuously being remodelled throughout life by a process of resorption and new bone formation. Bone consists of an organic matrix (osteoid), primarily composed of collagen, in which calcium phosphate crystals in the form of hydroxyapatite are deposited, to create the hard bone matrix. Osteoblasts, osteoclasts and osteocytes are key cell types involved in bone remodelling. Although calcitriol promotes bone calcium mobilisation, its overall effect is complex involving indirect stimulation of osteoclasts, decreased collagen synthesis by osteoblasts, but the net effect is to restore bone formation.

#### Deficiency disease

Deficiency of vitamin D results in defects in bone mineralisation leading to rickets in children and osteomalacia in adults. Rickets is characterised by growth retardation and bone deformities such as bow legs; and osteomalacia by softening of the bone, widespread bone pain and muscle weakness. The deficiency may be caused by poor dietary intake of vitamin D, inadequate exposure to sunlight, malabsorption and liver or renal disease resulting in ineffective formation of calcitriol. Also vitamin D deficiency is prevalent in obese individuals due to a defect in hormone storage. Osteoporosis is a disease characterised by a reduction in bone density leading to an increased risk of fractures, and is a major health issue in the elderly population. Bone density declines after midlife and the rate of loss accelerates in women after menopause due to loss of oestrogen. Vitamin D insufficiency is also often a contributing factor. Vitamin D replacement and calcium supplementation are a key part of treatment options for these conditions.

In addition to bone diseases, vitamin D deficiency has been linked with other conditions such as multiple sclerosis (MS). MS is a T cell-mediated chronic autoimmune disease leading to destructive demeylination of neurons. Symptoms are broad depending on which part of the nervous system is affected. Both genetic and environmental factors are thought to underlie the disease and epidemiological studies have identified a geographical disease distribution in which there is an increased incidence at latitudes further from the equator. A lack of exposure to the sun in early childhood and the consequent deficiency of vitamin D is thought to account for this. Several studies have shown that vitamin D has immunomodulatory effects on immune cells (B cells, T cells, macrophages) which may well be linked to MS risk.

#### Actions at molecular level

At molecular and cellular levels, calcitriol produces its effects by binding to its cytoplasmic target receptor (vitamin D receptor, VDR) (Figure 8). The receptor is a member of the nuclear receptor family (also known as ligand-activated transcription factors), which also includes receptors for steroid hormones (e.g. oestrogen, cortisol) and other fat-soluble vitamins such as vitamin A. The VDR forms a heterodimer with the retinoid X receptor (RXR) and this complex translocates to the nucleus where it binds to hormone response elements on DNA to regulate gene expression. In the intestinal epithelium it up-regulates the gene for the calcium channel (*TRPV6*), intracellular transporter calbindin D and a calcium pump to increase calcium uptake.

### Vitamin K and bleeding disorders

**Figure 9:**
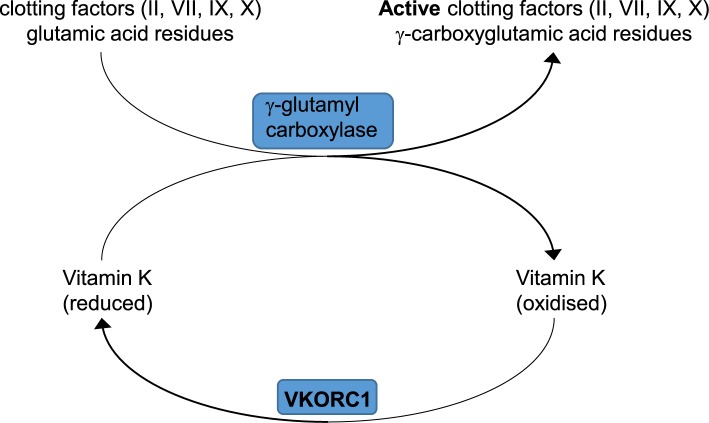
Vitamin K as a cofactor for γ-glutamyl carboxylation of clotting factors See text for details.

Vitamin K occurs in two biologically active forms – vitamin K_1_ (phylloquinone) and vitamin K_2_ (menaquinones). Both have a common ring structure (2‐methyl‐1,4‐naphthoquinone, also called menadione) and differ from each other in the length and saturation of the polyisoprenoid carbon chain attached at the 3 position. Vitamin K_1_ is abundant in leafy green vegetables (e.g. kale, spinach) while vitamin K_2_ is synthesised in various forms by bacteria present in the human intestine, and is also present in small amounts in fermented foods. Vitamin K is fat soluble and is absorbed with dietary lipids. It has an important function as a cofactor involved in formation of the active form of clotting factors involved in the coagulation cascade (factors II, VII, IX and X), and proteins involved in bone formation (osteocalcin). The reduced form of vitamin K acts a cofactor for the enzyme γ-glutamyl carboxylase which carboxylates glutamate residues in proteins, a modification which is required for calcium binding (Figure 9). During the reaction vitamin K is oxidised to its epoxide form and the enzyme vitamin K epoxide reductase complex 1 (VKORC1) converts it back again into its reduced form. The anticoagulant drug warfarin prevents blood clotting by inhibiting the VKORC1 enzyme, reducing recycling of vitamin K, and consequently the active form of blood-clotting factors (Figure 9). Deficiency of vitamin K in adults is rare but may occur as a consequence of disorders of bile flow (cholestatic jaundice) disrupting fat absorption or broad-spectrum oral antibiotic use which kills the gut microflora. In newborn babies, particularly premature infants, deficiency and consequently bleeding is a particular risk. Deficiency arises because placental transfer of vitamin K is poor, there is little vitamin K in breast milk and the gut of the newborn is essentially sterile restricting bacterial synthesis. As a consequence of the risk of bleeding in vitamin K deficient infants, supplementation is recommended shortly after birth.

### Obesity

The worldwide prevalence of obesity is reaching epidemic proportions. Over the past four decades, the prevalence of adult obesity has nearly quadrupled, with nearly 40% of the worldwide population classed as overweight, and rising numbers of children and adolescents affected. Once thought of as a problem of the developed world it is now also on the rise in low- and middle-income countries. The fundamental cause of obesity and overweight is an excess caloric intake coupled with a low level of physical activity; however, there are multiple contributing factors, some of which are discussed below. Being overweight, or obese, is defined as a disproportionate body weight for height and is generally calculated using the body mass index (BMI) (body mass (kg)/height (m)^2^), although this measure is recognised to be imprecise. The WHO classification defines normal values for BMI as 18.5–24.9 kg/m^2^; overweight as 25–29.9 kg/m^2^ and obese as ≥30 kg/m^2^.

Body weight, and consequently obesity, is determined by the complex interplay of energy intake and expenditure, psychosocial factors, genetic factors and by the body’s neural and endocrine systems. Since these aspects have been widely reviewed elsewhere (see ‘Further reading’ section), this section focuses on the interplay of environment and genetic factors, epigenetics, and some recent developments in understanding fat storage in adipocytes and pathological changes.

Studies examining the genetic basis of obesity in the wider population have identified hundreds of gene variants that are linked to obesity through physiological processes such as regulation of appetite control and energy metabolism, indicating that obesity is a polygenic disorder (i.e. it is associated with the effect of multiple genes), in combination with lifestyle and environmental factors. There are, however, a few very rare single gene variants that can underlie obesity. These genes include the leptin receptor gene (*LEPR*) which encodes for a hormone that is involved in long-term regulation of energy balance and suppressing food intake, and the melanocortin receptor 4 gene (*MC4R*) which is a receptor for the neurotransmitter α-melanocyte-stimulating hormone (α-MSH) in the hypothalamus of the brain, a region that controls appetite. It is clear that genes and the environment interact in complex ways to determine body weight variability. One interesting example comes from a study comparing genetically similar Pima Indians living either a traditional lifestyle in the Sierra Madre region of Mexico or in urban environments of Arizona with a westernised lifestyle. The levels of obesity in male U.S. Pima was almost ten-times higher than their Mexican counterparts, and similarly Type 2 diabetes was also five-times higher. The Mexican Pima have a high level of physical activity and a simple low-fat diet that is high in fibre; and certain genes may provide a protective or survival advantage in this environment when food is scarce. In westernised environmental conditions (low physical activity and diet high in fat and refined sugars), genetically the Pima have a predisposition to obesity although this is not inevitable and can be prevented by lifestyle change.

Another example of genetic and environmental interactions is provided by studies of medical records from the Dutch Hunger Winter in 1944. During the German occupation of the Netherlands, people were exposed to a period of severe famine and this was alleviated following liberation in May 1945. Babies born to mothers exposed to famine in mid to late gestation had a lower birth weight, remained small throughout their lives, despite the availability of food, and had lower obesity rates than the general population. On the other hand, babies born to mothers exposed to famine during the early gestational period had a normal birth weight, presumably because the fetus was able to ‘catch up’ in the later stages of development, but had higher obesity rates than normal later in life. Surprisingly some of these effects were also apparent in the grandchildren of the women who were malnourished. This intergenerational study indicates that nutritional deprivation can have long-lasting non-genetic effects on the body weight of offspring. It is thought that these changes are passed down via chemical modifications of DNA such as methylation, rather than sequence changes, which subsequently affect gene expression. Such changes are referred to as epigenetic modifications of the genome.

Recently much attention has been focused on the gut microbiota (i.e. the microrganisms resident in the gut) and links to obesity. Studies have demonstrated that the microbiota of individuals with obesity is less diverse than individuals with a normal weight. Also studies of the microbiota of twins discordant for obesity (i.e. they have a significant BMI difference) have demonstrated that the metabolic phenotype (i.e. normal or obese) could be transmitted to germ-free mice by transplanting the human faecal microbiota. The basis for the differential effects of microbiota on body weight are unclear but may be due to differences in microbial metabolism of fibre or other nutrients.

Adipocytes have important roles in both energy storage and as endocrine cells (i.e. they are able to secrete hormones and other factors). The storage function involves taking up fatty acids absorbed following a meal, or produced from glucose by the liver, and storing them as triglycerides. These long-term energy stores are broken down to release fatty acids during fasting or prolonged or intense exercise. The endocrine function involves sensing energy storage and releasing hormones such as leptin and adiponectin. Leptin reduces food intake and activates pathways causing lipolysis; while adiponectin improves insulin sensitivity, promoting glucose uptake, and ultimately fat accumulation when there is additional storage capacity. During periods of overnutrition the adipocytes increase in size and number to accommodate the need for fat storage; however, at a certain point the maximum capacity is reached and the stressed or damaged adipocytes start an inflammatory process that contributes to the health problems linked to obesity. The location of adipose deposits may either be abdominal, as typically seen in obese men, or subcutaneously in buttocks and thighs. These body shapes are often referred to as ‘apple-shaped’ or ‘pear-shaped’, and it is recognised that the former (excess abdominal fat) is more associated with a disease risk than other distributions.

Various mechanisms have been proposed as the trigger of the inflammatory response in adipose tissue that leads to increased production of the pro-inflammatory cytokines tumour necrosis factor α (TNFα) and interleukin 6 (IL-6). These include: a lack of oxygen supply to the adipose tissue (hypoxia), death of the stressed adipocytes leading to influx of monocytes which are transformed into resident macrophages that secrete the inflammatory cytokines; and mechanical stresses as the adipocytes interact with the dense extracellular matrix that they are embedded within.

Obesity is a major risk factor for conditions such as Type 2 diabetes, cardiovascular disease, certain types of cancer, non-alcoholic fatty liver disease and several other diseases. Non-alcoholic fatty liver disease is due to excess fatty acids being deposited in the liver which normally contains little or no fat. This condition can develop into more serious hepatitis and cirrhosis leading to liver failure. Obesity-associated cancer may be driven by the high levels of insulin and insulin-related growth factors, the chronic inflammatory environment or in the case of breast and ovarian cancers, by the fact the adipose tissue produces oestrogen, which can stimulate abnormal cell growth.

## Liver disease

### Alcoholic liver disease

Excessive alcohol intake is a common cause of liver disease and is associated with an increased risk of cancers and cardiovascular disease. The liver sustains the largest degree of tissue injury because it is the primary site of alcohol metabolism. Since the liver has the capacity to regenerate, symptoms of liver damage may not be apparent until damage is quite extensive. There are three main categories of alcohol-related liver damage: alcoholic fatty liver disease, alcoholic hepatitis and cirrhosis. The first category, a build-up of fat in the liver, occurs in almost all heavy drinkers and can occur even after a single drinking session. It is usually reversible on stopping drinking, and there are rarely any long-term symptoms. The second category, alcoholic hepatitis, which is distinct from infectious hepatitis, most often occurs in people who drink heavily over many years. It is characterised by inflammation of the liver with destruction of liver cells and some replacement of healthy tissue with scar tissue (known as fibrosis). This is a serious and life-threatening condition. Symptoms may include jaundice, nausea and vomiting, weight loss and fatigue. The more advanced form of the disease (cirrhosis) in which there is extensive fibrosis, in turn leads to liver failure and is often fatal. In addition to the liver, consumption of alcohol during pregnancy can have adverse effects on the developing foetus, referred to as foetal alcohol syndrome which is characterised by growth impairment, mental retardation and facial abnormalities.

**Figure 10:**
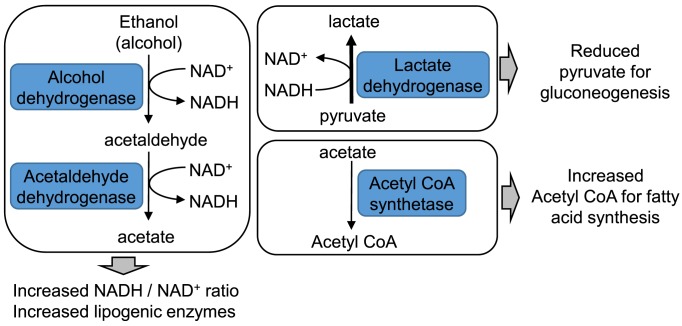
Ethanol metabolism and links with other metabolic pathways

Alcohol is metabolised in the liver by the enzyme alcohol dehydrogenase to acetaldehyde, and in turn this is metabolised by aldehyde dehydrogenase to acetate, which is released into the bloodstream. Both reactions use NAD^+^ as a cofactor, generating its reduced form NADH in the process. An alternative metabolic pathway is the enzyme cytochrome P450 2E1 (CYP2E1). Although this enzymatic pathway is considerably less efficient than alcohol dehydrogenase, because it is an inducible enzyme its role becomes more important in heavy drinkers due to its enhanced expression levels. This pathway also contributes to the development of tolerance (i.e. increasing levels of alcohol consumption are required to achieve the same effect). The enhanced generation of NADH by both enzymes increases the NADH/NAD^+^ ratio in liver hepatocytes, altering the cellular redox potential (i.e. balance of reactions involving reduction and oxidation) which favours formation of fatty acids and reduces gluconeogenesis (Figure 10). The enhanced formation of fatty acids is also due to an increase in expression of lipogenic enzymes, via effects on transcription factors, and conversion of acetate into acetyl CoA, a precursor for fatty acid synthetic reactions. Reduced gluconeogenesis can be explained as follows. Following conversion of glucose into pyruvate, during glycolysis, with the associated reduction of NAD^+^ to NADH, pyruvate is converted into lactate with the oxidation of NADH to NAD^+^. This latter reaction is promoted by the increased NADH/NAD^+^ ratio following ethanol metabolism resulting in reduced availability of pyruvate for gluconeogenesis. This effect together with poor nutrition contributes to the hypoglycaemia which is common in alcoholics.

Excessive alcohol intake also leads to inflammation of the liver (hepatitis). Inflammation occurs following activation of the macrophage immune cells that are normally residing in the liver, and infiltration of further macrophages. Normally the resident macrophages (Kupffer cells) provide a protective role against foreign pathogens; however, excessive alcohol exposure switches them to a pro-inflammatory state. Deposition of connective tissue (fibrosis) is also seen in liver cirrhosis. This stiffens blood vessels and damages the internal structure of the liver leading to severe complications and liver failure. Liver transplantation is an established procedure to remove an unhealthy liver and replace it with a healthy one from a donor. The procedure may be used for treatment of a number of liver diseases. A healthy liver may be obtained from an organ donor after death or, in some cases, part of liver may be obtained from a living donor, often a family member or friend.

## Neurological/neurodegenerative

### Alzheimer’s disease

Alzheimer’s disease is the most common neurodegenerative disease. It affects approximately 10% of the population over age 65 and 30% of individuals aged 85 or older. There are also much rarer cases in younger individuals (mean age: ∼45 years) that are often associated with an inherited form of the disease. Increasing age, genetics and head trauma are all recognised as risk factors for the disease. As with other forms of dementia, the symptoms may include impairment of memory, language, reasoning and visual perception.

**Figure 11:**
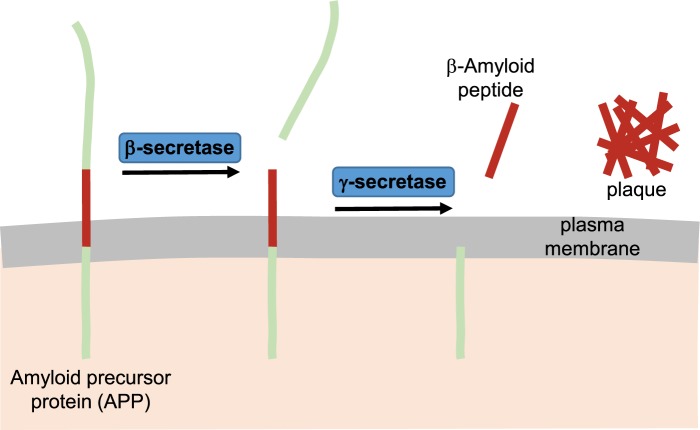
Pathogenesis of Alzheimer’s disease via β-amyloid See text for details.

The most widely accepted hypothesis for the molecular basis of Alzheimer’s disease is centred around β-amyloid peptides (Figure 11). In this pathway, β-amyloid is generated from amyloid precursor protein (APP) via sequential proteolysis by the proteases β-secretase and γ-secretase. The β-amyloid peptides of 40–42 amino acids in length spontaneously form aggregates which go on to form fibres that are deposited in plaques outside neurons in the brain. Deposition of these plaques is a characteristic of Alzheimer’s disease and it is these events that lead to neuronal degeneration and cognitive decline. Within neurons, the microtubule-associated protein τ becomes modified and forms aggregates that go on to form intracellular tangles (neurofibrillary tangles), a process leading to cell death. These plaques and tangles spread throughout the brain in a predictable pattern, typically originating in the hippocampus, limbic system, frontal and temporal lobes, and then progressing to other areas of the cortex in advanced disease.

The above hypothesis is supported by strong evidence from studies of genetic mutations in patients with early-onset Alzheimer’s disease. Mutations in the genes for APP and presenilins 1 and 2 (PSEN1, PSEN2) have been identified in many of these patients. The γ-secretase is known to be a complex of presenilins 1 and 2 and other proteins. These mutations are associated with an increased production of Aβ42 (the 42-amino acid β-amyloid peptide) that is more prone to aggregate and form plaques. Also in Down’s syndrome patients, where there is an extra copy of chromosome 21, which carries the *APP* gene, there is increased production of APP and these individuals all develop Alzheimer’s like disease. Conversely, a mutation in the *APP* gene has been identified that decreases APP production and provides a protective effect against development of Alzheimer’s disease.

Population studies have identified that the apoliprotein E4 (*APOE4*) gene is a risk factor for developing Alzheimer’s disease. Three common polymorphisms of the *APOE* gene exist (E2, E3 and E4) and multiple copies of the APOE4 increases disease risk significantly and are associated with an earlier age of onset of Alzheimer’s disease. The ApoE protein is known to play an important role in lipid transport (see above section on ‘Atherosclerosis’). Its role in the pathogenesis of Alzheimer’s disease is not clear but it is thought to have an effect on β-amyloid deposition and clearance, and ultimately plaque formation.

There are currently no effective cures, or even treatments that significantly inhibit the progression of the disease, although some treatments may temporarily improve the behavioural problems. One class of approved drugs known as ‘cholinesterase inhibitors’, act by preventing the breakdown of the chemical messenger acetylcholine in the brain. Acetylcholine is important for learning and memory and the drugs slow the breakdown of this molecule, which may help to compensate for the loss of function brain cells. Another drug memantine, works by regulating the activity of the neurotransmitter glutamate, which in excess is thought to have cytotoxic effects linked to Alzheimer’s disease. Many clinical trials have investigated the use of inhbitors of β-secretase and γ-secretase as a means to reduce β-amyloid production and slow cognitive decline; however, to date the majority of these trials have not demonstrated beneficial effects of the drugs.

## Footnotes

1 A biomarker is a naturally occurring molecule, gene, measurable physiological characteristic or process that is an indicator of a particular disease state.

## Abbreviations

APP: amyloid precursor protein
BCR: break-point cluster region
BMI: body mass index
CCR5: chemokine receptor type 5
CFTR: cystic fibrosis transmembrane conductance regulator
CML: chronic myeloid leukaemia
EGF: epidermal growth factor
GLUT-4: glucose uptake transporter type 4
HDL: high-density lipoprotein
LDL: low-density lipoprotein
MS: multiple sclerosis
oxLDL: oxidised LDL
PCSK9: proprotein convertase subtilisin/kexin type 9
PKU: phenylketonuria
PUFA: polyunsaturated fatty acid
VDR: vitamin D receptor
VKORC1: vitamin K epoxide reductase complex 1
VLDL: very low-density lipoprotein

## References

[B1] IDF Diabetes Atlas - 8th Edition (2017) http://diabetesatlas.org/resources/2017-atlas.html

[B2] Diabetes UK - The Cost of Diabetes (2014) https://www.diabetes.org.uk/resources-s3/2017-11/diabetes%20uk%20cost%20of%20diabetes%20report.pdf

[B3] Ahlqvist E., Storm P., Karajamaki A., Martinell M., Dorkhan M., Carlsson A. (2018) Novel subgroups of adult-onset diabetes and their association with outcomes: a data-driven cluster analysis of six variables. Lancet Diab. Endocrinol. 6, 361–369 10.1016/S2213-8587(18)30051-2 29503172

[B4] Rich S.S. (2016) Diabetes: still a geneticist’s nightmare. Nature 536, 37–38 10.1038/nature18906 27398618

[B5] Samuel V.T. and Shulman G.I. (2012) Mechanisms for insulin resistance: common threads and missing links. Cell 148, 852–871 10.1016/j.cell.2012.02.017 22385956 PMC3294420

